# Challenging the Myth of the Digital Native: A Narrative Review

**DOI:** 10.3390/nursrep13020052

**Published:** 2023-04-04

**Authors:** Lisa Reid, Didy Button, Mark Brommeyer

**Affiliations:** 1 College of Nursing and Health Sciences, Flinders University, Adelaide 5042, Australia; 2 Flinders Digital Health Research Centre, Flinders University, Adelaide 5042, Australia; 3 College of Business, Government and Law, Flinders University, Adelaide 5042, Australia; 4 College of Public Health, Medical and Veterinary Sciences, James Cook University, Townsville 4811, Australia

**Keywords:** nursing education, undergraduate curricula, nursing workforce, digital literacy, information and communication technologies, digital health

## Abstract

Background and Aims: Nurses are increasingly engaging with digital technologies to enhance safe, evidence-based patient care. Digital literacy is now considered a foundational skill and an integral requirement for lifelong learning, and includes the ability to search efficiently, critique information and recognise the inherent risk of bias in information sources. However, at many universities, digital literacy is assumed. In part, this can be linked to the concept of the *Digital Native*, a term first coined in 2001 by the US author Marc Prensky to describe young people born after 1980 who have been surrounded by mobile phones, computers, and other digital devices their entire lives. The objective of this paper is to explore the concept of the Digital Native and how it influences undergraduate nursing education. Materials and Methods: A pragmatic approach was used for this narrative review, working forward from Prensky’s definition of the Digital Native and backward from contemporary sources of information extracted from published health, education and nursing literature. Results: The findings from this narrative review will inform further understanding of digital literacy beliefs and how these beliefs influence undergraduate nursing education. Recommendations for enhancing the digital literacy of undergraduate nursing students are also discussed. Conclusions: Digital literacy is an essential requirement for undergraduate nursing students and nurses and is linked with safe, evidence-based patient care. The myth of the Digital Native negates the reality that exposure to digital technologies does not equate digital literacy and has resulted in deficits in nursing education programs. Digital literacy skills should be a part of undergraduate nursing curricula, and National Nursing Digital Literacy competencies for entry into practice as a Registered Nurse should be developed and contextualised to individual jurisdictions.

## 1. Introduction

Digital literacy is considered one of the foundational literacies for learning. The World Economic Forum [1] (p. 10) defines foundational literacies as representing how an individual applies “core skills to everyday tasks”. “Digital literacy looks beyond functional IT (information technology) skills to describe a richer set of digital behaviours, practices and identities” [2], which change across contexts and time. Ng’s [3,4] development of a digital literacy framework identified digital literacy as the result of intersecting technical, cognitive and socio-emotional dimensions. *Technical dimensions* include technical skills for using digital technologies in everyday living and learning [3,4]. *Cognitive dimensions* require the ability to critique digital sources, evaluate the suitability of software programs and understand the ethical and legal implications of using digital sources [3,4]. *Socio-emotional dimensions* entail the responsible use of the Internet and the promotion of safety and privacy [3,4]. Ng [3,4] asserts that underpinning these three dimensions is *critical literacy*, the ability to critically evaluate information with an understanding of the inherent bias in sources of information. Other models and frameworks of digital literacy have been proposed [5,6,7], but the overarching aim of developing digital competencies and lifelong learning skills in education remains of paramount importance [8].

However, many universities are yet to recognise digital literacy as an inherent part of foundational literacies; Murray and Perez [9] (p. 850) noted that “at most universities, digital literacy is either taken for granted or assumed to be at an adequate level rather than being assessed, remediated and amplified”. This deficit in digital literacy is further exacerbated by the disparity between institutional responses to digital literacy requirements [5] and the prevailing belief that students’ increased exposure and use of technology correlates with digital literacy [9,10,11]. In part, these beliefs can be linked to the concept of the *Digital Native*, a term created by Prensky [12] to describe students who have grown up with digital technology and “think and process information fundamentally differently from their predecessors”. 

Since Prensky’s seminal work [12], *Digital Natives*, *Digital Immigrants*, there has been debate over whether it presents a false dichotomy [10,13] that young people instinctively know how to use digital technologies as opposed to *Digital immigrants,* who are exposed to digital technologies later in life [12,14]. Debate has also centred around whether the ability to use mobile phones and other handheld devices equates with digital literacy and whether young people overestimate their digital competency [15]. Despite these arguments, the myth of the Digital Native is still evident in universities [3,14,16].

Therefore, this narrative review will examine the history of the Digital Native, the arguments for and against this terminology, and how these beliefs influence the digital literacy of undergraduate nursing students. Recommendations for enhancing the digital literacy of undergraduate nursing students are also discussed. 

## 2. Materials and Methods

A pragmatic approach was used for this narrative review, working forward from Prensky’s definition of the Digital Native and backward from contemporary sources of information extracted from the published health, education, and nursing literature. “Narrative reviews describe published articles to inform debate, appraise research and identify gaps in current knowledge” [17] (p. 109) and are the most common publications in medical literature [18,19]. “Narrative overviews are useful educational articles since they pull many pieces of information together into a readable format” and provide a broad perspective of a phenomenon of interest [20] (p. 103). It is important to note that despite differing from the methodological requirements of a systematic review, narrative reviews remain systematic and are not an ad hoc review [21]. This review, undertaken as part of a PhD research study, identifies the history of the Digital Native, thereby adding to the body of knowledge regarding digital literacy and undergraduate nursing curricula.

### 2.1. Narrative Review Methodology

In response to the lack of a consistent narrative review methodology, Baethge et al. [18] developed the *Scale for the Assessment of Narrative Review Articles (SANRA),* which consists of six items: (1) a justification of the article’s importance for the readership, (2) a statement of concrete/specific aims or the formulation of questions, (3) a description of the literature search, (4) referencing, (5) scientific reasoning, and (6) an appropriate presentation of data (as listed in Appendix A). These items are discussed below and applied to this narrative review.

#### 2.1.1. Item 1—Justification of the Article’s Importance for the Readership

Justification of the relevance and importance for the reader [18] is important. In this instance, a coherent discussion about the history of the Digital Native is provided. In this review, the arguments for and against this terminology and the ways in which these beliefs influence the digital literacy of undergraduate nursing students are articulated accordingly.

#### 2.1.2. Item 2—Statement of Concrete/Specific Aims or Formulation of Questions

This requires a clear statement of the aims or questions of the review [18]. The aim of this review is to provide an analysis of the Digital Native debate and provide recommendations for enhancing the digital literacy of undergraduate nursing students.

#### 2.1.3. Item 3—Description of the Literature Search

Here, a clear and transparent description of the search strategy, including search terms and the types of literature included in the search, is required; however, “it is not necessary to describe the literature search in as much detail as for a systematic review (searching multiple databases, including exact descriptions of search history, flowcharts etc.), but it is necessary to specify search terms, and the types of literature included” [18]. A detailed description of the search strategy is provided below.

##### Search Strategy

A literature search of English-language peer-reviewed and full-text articles was conducted using the search terms “*education AND nursing*”, “*digital literacy*” and “*Digital Native*”. Additional search terms of “*Net generation*”, “*Generation Y*” and “*Google generation*” were then applied, as identified by the ECDL (European Computer Driving Licence) [15] in “The Fallacy of the Digital Native.” Identified sources of information were included if they were published between January 2001 and January 2023 to reflect the period of time since the first use of the term *Digital Native*.

Inclusion criteria included: articles that were peer-reviewed and available as a full-text article, described Digital Natives (or equivalent definitions), related to digital literacy, related to undergraduate nursing education, were published between January 2001 and January 2023 and were published in English.

Exclusion criteria included: articles that were not peer-reviewed, not available as a full-text article, not related to Digital Natives (or equivalent definitions), not related to digital literacy, not related to undergraduate nursing education or nursing education, not published between January 2001 and January 2023, not available in English, duplicate articles and articles for which the authors were unable to access the full text.

##### Database

ProQuest Central was searched to identify potentially relevant sources, including scholarly journals, books, reports, conference papers and proceedings. A total of 4084 sources of evidence were identified. A snowball technique was employed, and 13 additional sources of information were obtained from the reference lists of selected sources. In total, 4097 sources of evidence were uploaded to Covidence, an online collaboration platform that facilitates the preparation of literature reviews, aids in uploading search results, the screening abstracts and full texts, completing data collection, review by two or more reviewers and exporting data [22]. Following duplicate removal, 3837 sources of evidence progressed to the screening process.

##### Screening Process

**Figure 1:**
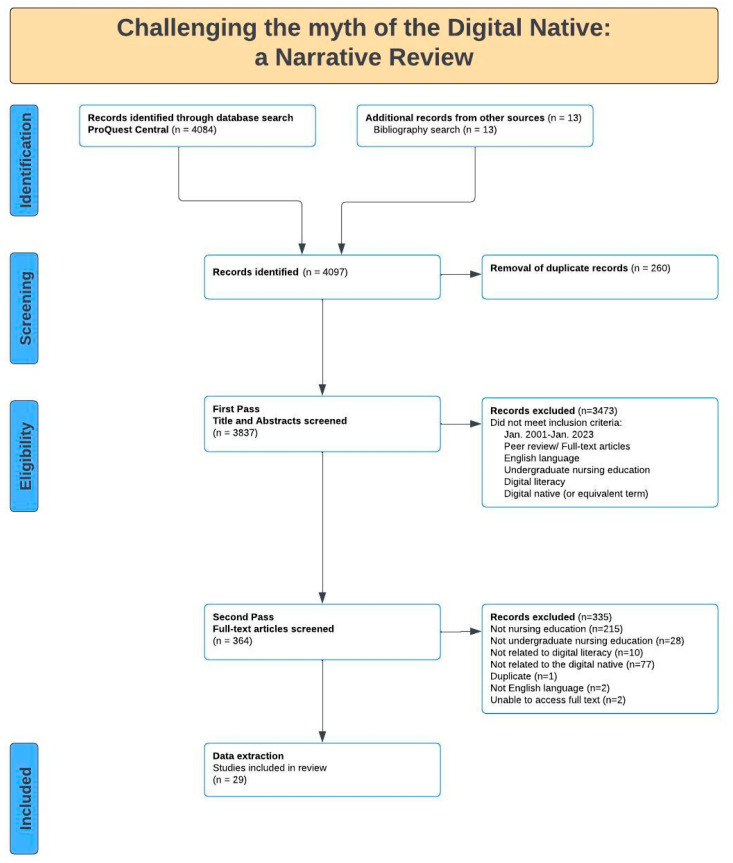
The reporting items for the screening processes used in this narrative review.

The screening process determined whether each source met the inclusion criteria. The screening process involved *Title and abstract screening* and *Full-text screening*. A total of 3837 sources of evidence progressed to *Title and abstract screening*, and 364 sources of evidence progressed to *Full-text screening*. To arrive at a consensus, review meetings were held, and emails were exchanged between the researcher and the PhD supervisors. For visual reinforcement and to enhance the trustworthiness of the findings, a flowchart was developed to depict the phases of the screening process (see Figure 1).

##### Data Extraction

Following the completion of the screening process, 29 sources of evidence were moved to the *Data extraction* phase, and a template was developed in Covidence with consultation between the researcher and the PhD supervisors. The template included the following headings: study details (title and authors), study settings, aim or purpose of the study, study design, sampling procedure and a synopsis of content related to the inclusion criteria. The data extraction template was used in the *data analysis and synthesis* phase.

##### Data Analysis and Synthesis

In a narrative review, the analysis and synthesis of data requires all the information retrieved in the literature search to be synthesised into comprehensive paragraphs [20]. Green et al. [20] recommend the use of a clear and systematic approach that identifies the relevant content and provides a discussion of major areas of agreement and disagreement. The author’s interpretation of selected sources of information should be provided with recommendations on the relevance of the findings [20]. The data analysis and synthesis with recommendations are provided in the Section 3, Section 4 and Section 4.4.

#### 2.1.4. Item 4—Referencing

Comprehensive referencing, including evidence for all arguments stated in the review, supports the validation of the trustworthiness of the findings [18]. Referencing for all sources used in this review is provided in the *References* section.

#### 2.1.5. Item 5—Scientific Reasoning

This item requires evidence for arguments, study designs of selected sources of information and, where applicable, levels of evidence [18]. Evidence for arguments is provided in the Section 3 of this review.

#### 2.1.6. Item 6—Appropriate Presentation of Data

The final requirement is concerned with the correct presentation of data from the selected sources of information [18]. Accordingly, appropriate conventions are applied to ensure the data are presented clearly and comprehensively in this review.

### 2.2. Narrative Review Definitions

#### 2.2.1. Definitions of Undergraduate Nursing Students

To be included in this review, sources of evidence needed to include education for undergraduate nursing students in a Bachelor of Nursing program (or equivalent). The Australian Nursing and Midwifery Federation [23] define undergraduate nursing students as individuals enrolled within a recognised nursing program leading to registration as a Nurse. To meet the requirements for registration as a Registered Nurse in Australia, individuals are required to complete a Bachelor of Nursing program at a university (Australian Qualifications Framework Level 7), as defined by the Australian Qualifications Framework Council [24,25].

#### 2.2.2. Use of Digital Technologies in Undergraduate Nursing Education

Digital technologies used in undergraduate nursing education were explored in the sources of evidence. Cremin [26] (p. 153), an eminent educational historian, defined education as “the deliberate, systematic, and sustained effort to transmit, evoke, or acquire knowledge, attitudes, values, skills, or sensibilities, as well as any outcomes of that effort”. Digital technologies are “electronic tools, systems, devices and resources that generate, store or process data. Well known examples include social media, online games, multimedia and mobile phones” [27].

#### 2.2.3. Digital Literacy Defined

Digital literacy has been defined as “the ability to use information and communication technologies to find, evaluate, create, and communicate information, requiring both technical and cognitive skills” [28]. “Digital literacy looks beyond functional IT skills to describe a richer set of digital behaviours, practices and identities. What it means to be digitally literate changes over time and across contexts, so digital literacies are essentially a set of academic and professional situated practices supported by diverse and changing technologies” [2]; however, there is no consensus on the definition [13].

#### 2.2.4. The Digital Native Described

In 2001, Prensky [12] first used the phrase *Digital Native*, stating “Our students today are all ‘native speakers’ of the digital language of computers, video games and the Internet”. In a subsequent publication, Prensky suggested that the brains of Digital Natives could also be physically different due to the input of digital technologies [29]. The concept of specific attributes of different generations and their engagement with digital technologies is not new [30], with Tapscott [31] identifying the *Net Generation* in 1998. However, Prensky’s *Digital Native* gained traction, particularly in academia, and has been present in the literature and public discourse ever since [15].

#### 2.2.5. Digital Native Assumptions

*Digital Natives* were defined by Prensky [29] as anyone born after 1980; however, time specifications for generations differ between researchers [32], with *Generation Y* or *Millennials*, born between 1981 and 1995 [33], *The Net Generation or Net Set,* born between 1980 and 2001 [34], *the Google Generation,* born after 1993 [35], and *Generation Z,* born between 1996 and 2010.

## 3. Results

Twenty-nine sources of evidence were selected following full-text screening and data extraction. The characteristics of these sources and their relevance to the inclusion criteria are presented in Table A1 (see Appendix A) and described below.

### 3.1. Undergraduate Nursing Education

#### 3.1.1. Definitions of Undergraduate Nursing Students

For the purpose of this review, undergraduate nursing students were defined as individuals undertaking a three-year Bachelor of Nursing program at a university. Equivalent definitions were also identified in the sources of evidence, including Bachelor of Science Nursing [36,37,38], nursing degree students [39], nursing students [40,41,42,43], baccalaureate nursing students [44,45] health professional students (including nursing) [46,47,48], undergraduate pre-registration nursing students [49], Generation Z, Net Generation or Millennial students (nursing) [50,51], undergraduate students (nursing) [52], university education (nursing) [53] and students (nursing) [54].

#### 3.1.2. Use of Digital Technologies in Undergraduate Nursing Education

For the purpose of this review, digital technologies used in (or recommended for use in) undergraduate nursing education were identified. They included computer-based or device-based applications [36,37,38,43,48,51,52,55,56,57], the Internet [36,37,41,44,48,54,55,58], social media platforms [36,37,39,42,48,50,51,53,56], learning management systems [36,37,56], online videos [32,39,46,48,50,51], online learning [37,51,53], e-portfolios [36], electronic health records and medication records [37,55,57], clinical simulations [40,43,51,59,60] virtual learning environments [39,43,56], interactive gaming [39,40,50,56,60,61], lectures with response clickers [44,59], blogs [59] and wikis [59].

#### 3.1.3. Faculty Responses to Digital Technologies in Undergraduate Nursing Education

Faculty knowledge has been identified as a barrier to the integration of digital technologies into undergraduate nursing curricula [62]. The knowledge, skills and attitudes of nursing faculty regarding digital technologies were highlighted in the sources of evidence as contributing to a lack of technology use in nursing education [37,38,47,54,55,56,57,59,60]. These factors were attributed to a lack of professional development in the use of digital technologies [37,55], a lack of confidence when using digital technologies [47], a lack of understanding of the role of digital technologies in nursing care [55], tension between technology- and human-based care [55] and an adherence to traditional approaches [38,52,54,56,59,60]. However, the potential for faculty to respond to the challenges of digital technology use in undergraduate nursing curricula was also identified, with recommendations for improving student engagement through embracing digital education strategies [37,39,40,42,43,44,47,50,51,55,56,57,59,60,61,63].

### 3.2. Digital Literacy

#### 3.2.1. Definitions and Relevance of Digital Literacy

Throughout the sources of evidence, digital literacy was consistently identified as a critical component of success [38]. Some sources of evidence equated the increased exposure to digital technologies, experienced by those born after 1980, as meeting digital literacy requirements [32,40,59,60,61]; however, caution against generalising about the digital literacy of a generation was identified [43,46,53]. It was also noted that access to information through electronic media, whilst often equated with digital literacy, has resulted in “a weakness in critical thinking and a lack of understanding of the differences between true, objective facts versus opinions” [44] (p. 160), with those ill-prepared for the use of digital technologies being subject to reality shock as they enter the workforce [27]. Sub-sections of digital literacy identified in the sources included eHealth literacy [47,63], computer literacy [37], digital information literacy [64], communication literacy [28], online information literacy [58] and media literacy [48].

#### 3.2.2. Development of Digital Literacy in Undergraduate Nursing Education

The discussion on the development of undergraduate nursing students’ digital literacy was noted to be limited in some of the sources of evidence. As previously illustrated, some authors made assumptions about generational differences and inherent digital literacy, and therefore subsequent development of digital literacy skills was not addressed. However, studies identified the importance of promoting critical thinking and clinical reasoning [39,40,46,47,56,58,60,63,64], the development of a professional digital identity [36] and the development of digital technology skills for the workforce [43,54,55,56].

### 3.3. The Digital Native

#### 3.3.1. Descriptions of the Digital Native

In a large proportion of the sources of evidence, the term *Digital Native* [37,38,39,42,43,44,46,48,49,51,52,53,54,55,57,58,59,63] or an equivalent term was used, including *Generation Y* [32,36,45,46,60], *Millennials* [32,39,40,41,50,52,59,61], *Net Generation* [32,52,53,60], *Net Set* [52], *Google Generation* [32] and *Generation Z* [36,39,40,41,43,44,58]; however, Mather et al. [47] avoided the use of these terms, referring to *the next generation*.

#### 3.3.2. Digital Native Assumptions

Assumptions about the specific attributes of different generations and their engagement with digital technologies were evident in a number of sources of evidence. *Generation Y* was described as “a unique and truly Digital Native generation” [56] (p. 180), with the “ability to obtain instantaneous results” [36] due to access to digital devices and the expectation of immediacy in responses and information. *The Net Generation* was described as having unique learning styles [60] and information literacy [54]. *Generation Z* was identified as being uniquely diverse, tech-savvy and self-motivated [50], hyperconnected to digital technologies [44] and comprising true Digital Natives [56]. Overall, *Digital Natives* were noted to require flexible, collaborative and individualised learning [53], were confident in the use of digital technologies [64] and sought electronic resources for accessing health-related information [63].

#### 3.3.3. Digital Native Criticisms

The debate surrounding the Digital Native has been described as an academic form of a moral panic, with suggestions that the education system must be fundamentally changed to meet the need of a new generation of students [65]. Brown and Czerniewicz [66] noted that one of the major issues with the terminology was the creation of a false dichotomy or binary opposition between those who were considered natives and those who were not. Similarly, some of the sources of evidence highlighted the problematic nature of the Digital Native narrative. Walker et al. [45] found no statistical differences between the learning and teaching needs of Generation X and Generation Y students. Hills [46] (p. 15), in a systematic review of *Generation Y Health Professional Students’ Preferred Teaching and Learning Approaches*, concluded that the review could “neither confirm nor refute taking a generational perspective to explore teaching and learning preferences” and noted that “Preferences among generational groups were not consistent”.

## 4. Discussion

Evans and Robertson [67] agreed that the Digital Native debate is still evolving, and that there is no easy answer as to whether the Digital Native exists or not because the findings remain inconsistent. From a sociological perspective, some scholars argue for more technology for learning, while others argue for less use to improve wellbeing [68,69].

Regarding nursing education, this review found that over 22 of the 31 articles reviewed were still using the term digital native [37,38,39,42,43,44,46,48,49,55,57,58,63]. A 2022 study by Janschitz and Penker [70] confirmed that higher education students cannot directly transfer their digital skills to their course studies. They also found that females leaving school were “low digitised” compared to their fellow male students, a finding that needs to be further considered by nursing education programs in which female students predominate.

The results evidenced that nursing education is embracing a wide variety of digital technologies to meet the learning needs of students. However, the review also found that nursing educators are still not being supported with appropriate, accessible professional development opportunities to overcome their lack of confidence in using digital technologies in their teaching [37,40,55]. These identified barriers are compounded by the limited digital literacy skills of undergraduate nursing students when commencing higher education. Students’ continuing lack of digital literacy further repudiates the myth of the Digital Native and supports the findings of Walker et al. [45] that there is no firm evidence to support learning style preferences. 

There was confusion among some of the authors of the reviewed articles who incorrectly assumed that increased exposure time to technology equated to increased digital literacy skills [32,40,53,60,61]. In contrast, there were authors who championed the need to develop critical thinking and clinical reasoning skills above the development of digital literacy skills alone to provide a safe, literate workforce [39,40,42,46,47,50,51,54,56,58,60,63,64]. This would then suggest that the role of the digital native and the effect of digital literacy skills for undergraduate nursing students necessitates consideration of the assumed correlation.

### 4.1. The History of the Digital Native

In sociology, Generational Theory was developed as a mechanism to explain differences between population cohorts [71]. It was most notably described by Mannheim [69,72], a German sociologist in the 1950s. Mannheim asserted that the study of generations provides a means of understanding society, and that generations were primarily formed through a common location in history with shared experiences and events. Subsequent Generational Theories highlighted the importance of understanding generational differences to facilitate social change [72] and as a tool to decode reality [73], with Ryder [72] (p. 40) describing “the succession of birth cohorts (a construct similar to Mannheim’s formulation of generations) as a process of lending flexibility and providing new perspectives to address social problems”. In 1998, Tapscott [31] (p. 2) published *Growing up digital: The rise of the net generation*, stating “…it is through the use of digital media that the N-Generation will develop and superimpose its culture on the rest of society…they are a force for social transformation”. Several years later, Prensky [12] published *Digital Natives*, *Digital Immigrants* and exclaimed that there was a radical change seen in the students of today, declaring that they were no longer compatible with the education system designed to teach them. As the literature conceptualised a generation who inherently knew how to use digital technologies to such an extent, it was suggested this could result in physical changes in the brain [12,29]. This was embraced in public discourse [15], resonating with teachers, parents and policy-makers [30] and, despite debate in academic circles, became part of the cultural lexicon [74,75,76]. 

#### 4.1.1. The Digital Native Debate

Since the term *Digital Native* was first described in 2001 [12], the metaphor has been debated. Prensky [12] described this population as “all ‘native speakers’ of the digital language of computer, video and the Internet”, with multi-tasking, parallel-thinking abilities and a lack of patience for traditional learning approaches [12,29,77]. The concept of the Digital Native has been cited in many studies since this time [3,54,65,66,76] and continues to be mentioned in contemporary literature [53,78,79,80,81]. Criticisms of the Digital Native metaphor have pointed to a lack of empirical evidence in Prensky’s work [11,65,66,75,82], the assertion that exposure to digital technologies correlates with digital competence [11,15,82], overly emotive language [30,65,82], a false dichotomy between generations [15,65,75,83] and recommendations to abandon traditional teaching methods [11,65,81,83].

In 2009, Prensky [84] moved away from the Digital Native terminology to *Digital Wisdom*, indicating that as generations increasingly move into the 21st century, everyone will have grown up with digital tools and technologies, blurring the distinction between Digital Natives and digital immigrants. He also acknowledged that digital literacy and the ability to critique and evaluate digital technologies was an essential skill [15]. One issue often overlooked in the Digital Native debate has been the *Digital Divide*, described as the gap between those people with access to easy-to-use digital technologies and the Internet and those without this access [85]. Populations without access to these technologies include rural residents [85,86], low-income households [85,87], people with lower levels of education [85,88] and those from developing nations [87,89,90], with this lack of access identified as a human rights and social justice issue [85]. Despite these factors, the Digital Native rhetoric has persisted, with the continued promotion of this vocabulary having many beneficiaries, including those with commercial interests [74], and providing an unrealistic and ill-informed foundation for developing appropriate policy making and practice [91].

#### 4.1.2. Higher Education Responses to the Digital Native Debate

The responses by tertiary institutions to the Digital Native debate have been mixed. Smith [82], in noting the popularity of the Digital Native discourse, observed that despite “a growing body of recent evidence challenging such notions of students as digital natives”, there remained a dominant perception within higher education of the Digital Native generation. Burton [92] noted that the myth of the Digital Native, the belief in the internet as a “panacea” for rising education costs and demands for authentic learning experiences resulted in the widely held assumption that online learning was a quick, inexpensive and effective way of teaching. However, fundamental changes are required at an institutional level for effective online education to be realised. Other research noted that when educators assumed students to be Digital Natives, a note of caution was required for digital competence must be developed, not assumed [93]. A more nuanced approach was recommended which better informed and reflected the higher education and technology issues facing the current generations [82,94], with Bennett [94] (p. 329) stating that it was “time to move beyond the ‘digital natives’ debate as it currently stands, and towards a more sophisticated, rational debate that can enable us to provide the education that young people deserve”. This required the consideration of digital literacy skills among this cohort and among learners more generally.

### 4.2. Digital Literacy

#### 4.2.1. Defining Digital Literacy

As innovations in digital technologies have evolved, the language used to describe the knowledge, skills and attitudes required to use these technologies has also changed [13]. Boechler et al. [13] in Digital Literacy Concepts and Definitions: Implications for Educational Assessment and Practice, observed the evolution of these literacies from computer literacy, information literacy, and network literacy to digital literacy (knowledge and skills), with further development including a range of sub-categories such as e-literacy, digital competence and multimodal literacies. Alexander et al. [5] noted that definitions of digital literacy were nebulous, requiring greater clarification, and identified three different digital literacies: *universal literacy*—a baseline literacy embracing a critical stance towards all digital technologies; *creative literacy*—emphasising the technical skills of digital content production; and *literacy across disciplines*—a diffusion of digital literacy across the education curriculum which reflects different learning contexts. Digital literacy remains a contested concept, and its use has been inconsistent in the literature [95,96]. Digital literacy definitions have included “…socially situated practices supported by skills, strategies, and stances that enable the representation and understanding of ideas using a range of modalities enabled by digital tools” [97] (pp. 66–67), “the ability to use digital technologies—both hardware and software—safely and appropriately” [98] (p. 3) and “those capabilities which fit an individual for living, learning and working in a digital society” [2]. This presents challenges in being able to agree on a common lexicon.

#### 4.2.2. Institutional Responses to Digital Literacy

##### WHO—World Health Organization

Globally, institutional responses to digital literacy have been diverse. In Global diffusion of eHealth: Making universal health coverage achievable, the World Health Organization (WHO) [99] identified barriers to the global use of eHealth and acknowledged the need for a digitally literate health workforce, with the use of digital technologies in education recognised as a foundational element for training healthcare workers. Key factors associated with sustaining digital learning and educational transformation include recognising the current challenges of insufficient health worker competence, a lack of access to information and poor adherence to guidelines. The resultant recommendations included the “digital provision of training and educational content for health workers under the condition that it complements rather than replaces traditional methods of delivering continued health education and in-service training” [100] (p. 75). 

##### Jisc—Formerly the Joint Information Systems Committee

Established in 1993, Jisc is a not-for-profit digital, data and technology agency providing support for higher education institutions within the United Kingdom [101]. The agency provides useful advice by creating several guides to support the strategic development of digital literacies in higher education and identifying the seven elements of digital literacies that have applicability across all higher education teaching, including undergraduate nursing. The seven elements should be purposefully considered by all educators, as follows:

1. *Information Literacy*—the capability to find, critique and manage information;
2. *ICT Literacy*—the capability to adopt, adapt and use digital technologies;
3. *Learning Skills*—the capability to learn and study in a digital technology environment;
4. *Digital Scholarship*—the capability to participate in academic, research and professional environments that use digital technologies;
5. *Media Literacy*—the capability to critique and create academic and professional information using digital technologies;
6. *Communications and collaboration*—the capability to participate in digital environments for education and research;
7. *Career and identity management*—the capability to develop and manage a professional digital identity [98].

##### NMC—New Media Consortium

Since 2004, the New Media Consortium (NMC) has been responsible for publishing the Horizon Reports [102], which result from expert panel discussions and evaluations of contemporary trends in educational technologies. These reports are seen as valuable by the higher education sector, are cited in academic literature and have the potential to influence pedagogical approaches [103]. The first Horizon Report was published in 2004, a short time after the release of Prensky’s seminal work [12] *Digital Natives*, *Digital Immigrants*. A recent Horizon Report on higher education identified the “solvable challenge” of improving digital literacy, noting the current deficits in the promotion of digital literacy in higher education and advocating for the changing roles of educators to have more personal connections with students [104]. However, in A critical assessment of the NMC Horizon reports project [103], it was noted that New Media Consortium (NMC), responsible for publishing the Horizon Reports, had utilised a panel of experts including digital technology companies such as Apple Computer, Sony, Macromedia and Adobe Systems. The contention was that the information promulgated by the membership could have a leaning towards positive technological instrumentalism and the implicit assumption that technology is always better.

#### 4.2.3. Higher Education Responses to Digital Literacy

Whilst it has long been acknowledged that students require digital literacy skills to effectively engage with digital technologies [8,9,98,105], many higher education institutions have not adequately recognised digital literacy as one of the foundational literacies [9]. Murray and Perez [9] (p. 95), in their discussion on the digital literacy paradox in education, warned that exposure to digital technologies was not sufficient for developing digital literacy, and that “comprehensive digital literacy strategies that reach back to the youngest students and ensure that college graduates enter the workforce armed with these critical competencies” were an urgent need. The need for comprehensive and explicit digital literacy education to develop the necessary skills for the construction of learning is prefaced on the understanding that access to information cannot be seen as equivalent to access to knowledge [8]. The development of digital literacy within higher education requires “an institution-wide approach to building information, digital and data literacy skills”, thereby strengthening high-quality learning experiences [6]. It is therefore important to acknowledge the digital competence required for teaching and learning in undergraduate nursing.

### 4.3. Implications of the Digital Native Narrative on the Digital Literacy of Undergraduate Nursing Students

As the largest healthcare workforce [106], nurses need to embrace digital technologies to effectively function within the contemporary healthcare environment [107,108,109]. Theron et al. [110] (p.154) observed that “nurses use information and knowledge to inform practice and to educate individuals, families and communities with information that will assist them in making healthcare decisions that will positively impact their quality of life”. Therefore, knowledge and understanding of digital information are necessary for undergraduate nursing curricula to prepare graduates for an increasingly digital workplace [111,112,113,114]. As Brown et al. [109] (p. 457) observed, “It is imperative that curricula are developed and implemented so that students’ pre-existing and everyday digital literacy can be further developed, enhanced, and transposed to the bedside”. The failure to recognise digital literacy as a foundational competency, and the focus on the Digital Native are impeding the essential development of these necessary workforce skills.

### 4.4. Recommendations

Digital literacy is an essential requirement for undergraduate nursing students as they prepare to enter the workforce. However, this review has demonstrated that the continued dissemination of the myth of the Digital Native, which is accompanied by the perception that students arrive at university with digital literacy capabilities, is impacting students’ abilities to search efficiently, critique information and recognise the inherent risk of bias in information sources. From this study, the following recommendations are proposed:

1. A global set of core Nurse Educator Digital Literacy competencies are identified that can be contextualised to individual jurisdictions;
2. National Nursing Accreditation agencies adopt and contextualise National Nurse Educator Digital Literacy competencies and require all nurse academics to demonstrate their digital literacy competency accordingly;
3. Nurse Educator Digital Literacy competencies are recognised and aligned with existing national digital health competency frameworks;
4. National Nursing Digital Literacy competencies for entry into practice as a Registered Nurse are developed and adopted and are cognisant of the existing global efforts and frameworks to inform undergraduate nursing curricula;
5. National Nursing Accreditation and registration agencies update undergraduate course accreditation guidelines that reflect the development and assessment of the National Nursing Digital Literacy competencies.

### 4.5. Potential Barriers to Implementation

Implementing the five recommendations above could involve addressing potential barriers, including (1) systemic, with jurisdictional policy and regulatory hurdles to tackle; (2) organisational, with challenges faced in resourcing and a supportive change environment; (3) professional, with challenges in leadership and the recognition of a digital-first mindset; and (4) individual, with required changes in work practice reform and a supportive work environment [115].

### 4.6. Limitations

It is important to note some limitations associated with a narrative review. The researchers have a background in nursing education and health care management and are based in Australia. The focus on ProQuest Central as the searched database, in addition to the use of the snowball technique to identify additional publications from the reference lists of selected sources as part of the literature search, limits the generalisability of the results, though it is in line with the narrative review approach taken [18]. Therefore, the inclusion of further databases, such as Scopus, PubMed and ERIC, may generate additional publications relevant to this field of enquiry. The recommendations must then be considered in light of these limitations and contextualised to individual jurisdictions and environments.

## 5. Conclusions

This review has shown the dilemma facing today’s students and educators when relying on assumptions about digital capabilities, which can unwittingly perpetuate the myth of the Digital Native. The implications of this are profound for undergraduate nursing education.

The literature presented in this review supports the contention that digital literacy is an essential requirement for undergraduate nursing students and nurses and is associated with safe, evidence-based practice. The myth of the Digital Native presents a challenge to educators and curricula alike, as exposure to digital technologies does not necessarily equate with digital literacy. This assumption must be continually tested to ensure that nursing education programs are reflective of required practice in a digital world.

The five recommendations established from this research should inform future discussions and studies that investigate, substantiate and further encourage discourse throughout nursing education and digital health community. Digital literacy skills must be a part of undergraduate nursing curricula.

## Figures and Tables

**Figure A1:**
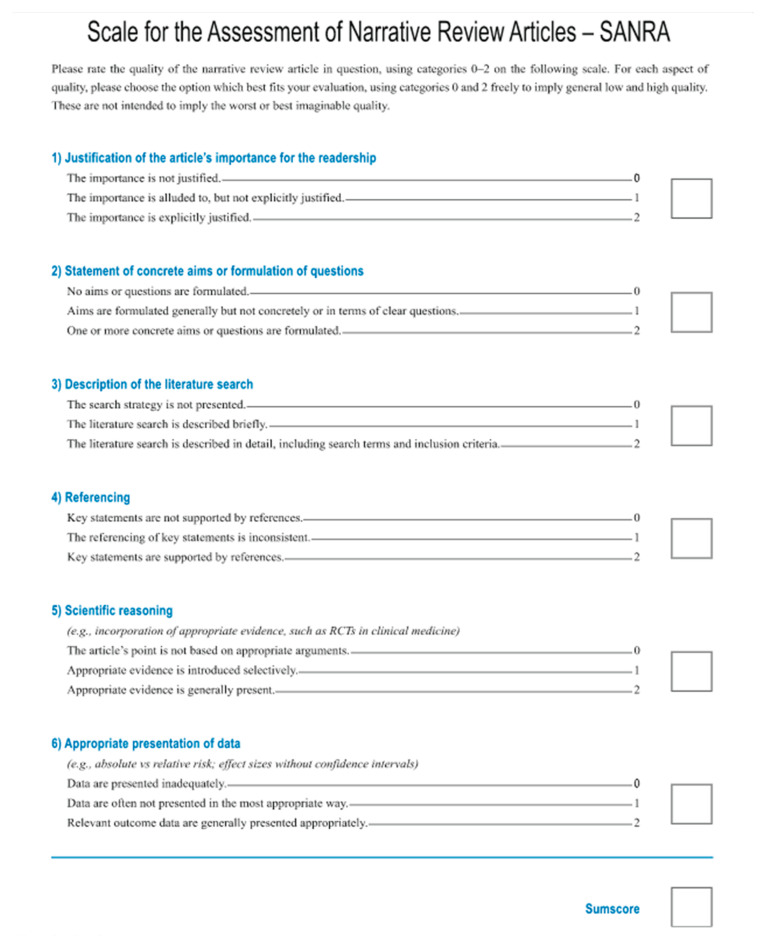
SANRA—Scale.

**Figure A2:**
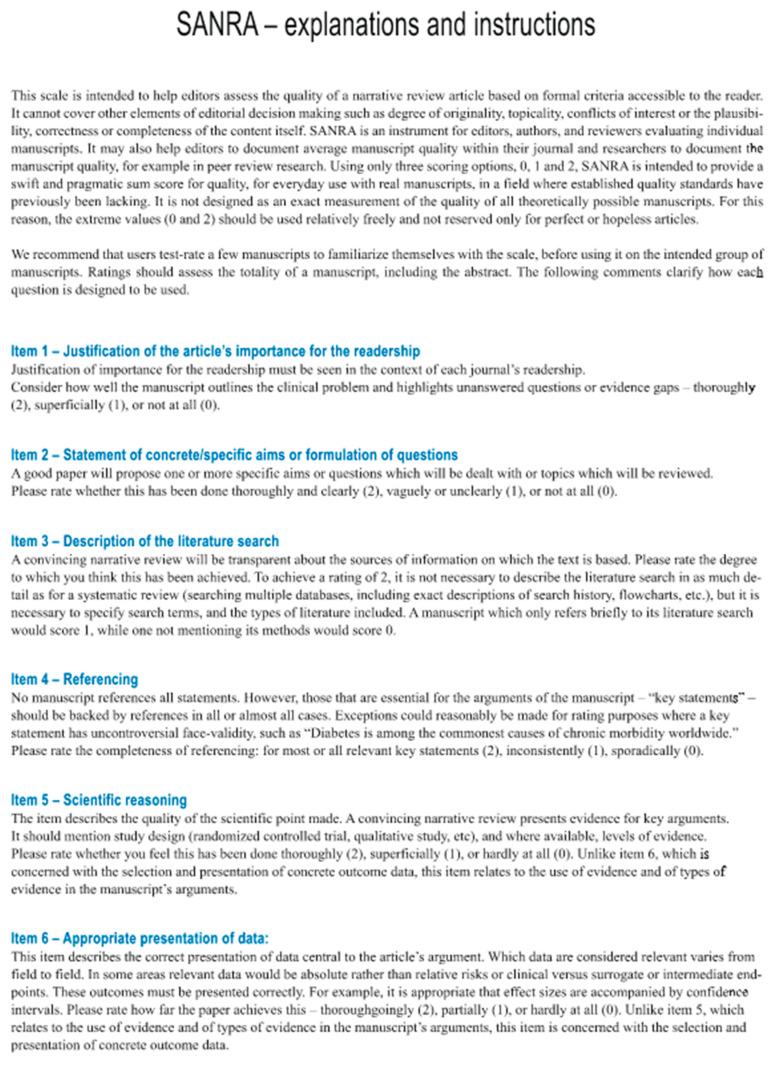
SANRA—explanations and instructions document.

## Data Availability

No new data were created or analysed in this study. Data sharing is not applicable to this article.

## Appendix A

**Table A1:** Data extraction of 29 relevant studies.

Number	Authors	Title	Country	Aim of Study	Study Design	Synopsis
1.	Walker et al., 2006 [45]	Generational (age) differences in nursing students’ preferences for teaching methods	United States	“This quantitative, descriptive research begins to examine the preferences and expectations of these generations regarding teaching methods.”	Descriptive survey	Provides in-depth definitions of generations with discussion of the differences in learning styles. Survey examined preferences for lectures, group work, case studies, web-based learning, self-directed learning and motivation for learning. No statistical significant differences between the preferences for Gen X and Gen Y students. Findings indicated “strong preference for faculty to structure the classroom and provide guidance, while indicating significant levels of trust in faculty to tell them what to do.” This is not supported by findings from a previous study.
2.	Mangold 2007 [61]	Educating a New Generation: Teaching Baby Boomer Faculty About Millennial Students	United States	“This review examines the impact of generational influences on the faculty-student relationship”	Literature review	Literature review regarding Millennial nursing education preferences and needs. In-depth definition of Baby Boomers and Millennials. Identifies learning preferences and the implications for nursing education, focusing on simulations, mentoring and research. “Traditional approaches to delivering nursing education do not fit the needs and desires of today’s student and tomorrow’s workforce. Faculty are playing a vital role in recruiting the next generation of nurses as they strive to reinvent the learning environment and themselves.” Discussion of the Digital Native—focuses on the differences between Baby Boomers and Millennials.
3.	Johnson et al., 2005 [40]	Generational Diversity: Teaching and Learning Approaches	United States	To provide readers with an overview of Generational Descriptions and Learning Characteristics	Scholarship of teaching	The article provides nurse academics a topic for a faculty/administrative workshop or discussion that enables faculty to expand upon the ideas presented here and develop context-appropriate teaching methods that address the generational diversity in their nursing courses.
4.	Hampton et al., 2020 [44]	Learning Preferences and Engagement Level of Generation Z Nursing Students	United States	“The purposes of this study were to identify the teaching methods that Generation Z nursing students preferred and felt were the most engaging and effective for learning and to determine their engagement level in the classroom setting.”	Cross-sectional study	Study of preferred teaching methods for Gen Z students. Shorter attention spans, weaknesses in critical thinking and working things out for themselves, lack understanding of the differences between true, objective facts versus opinion, reduced personal connections, prefer involvement in learning rather than lectures, want instant feedback. Discussion of the Digital Native: defines Gen Z and their learning needs and is linked with the digital native.
5.	Chicca et al., 2018 [56]	Connecting with Generation Z: Approaches in Nursing Education	United States	“This article identifies generational influences and distinctive characteristics of this group, which may challenge nurse educators and require changes in teaching and learning design strategies and approaches.”	Literature review	Discussion of Gen Z as “a unique and truly digital native generation”. “identifies generational influences and distinctive characteristics of this group”. Provides recommendations for educational change to meet learners’ needs, including augmenting traditional pedagogical practices with digital technologies. Recommendations for changes to the learning space. Also advises that students may be “lacking knowledge in how to perform some traditional adult activities and may even expect those in higher education to assume a parent-like role, or they may allow parents to continue to direct their development.” Discussion of the Digital Native: in-depth discussion of digital natives, millennials and Gen Z.
6.	Shatto et al., 2016 [50]	Moving on from Millennials: Preparing for generation Z	United States	“This article discusses the unique learning characteristics of Generation Z and de-scribes innovative teaching strategies to engage this new breed of student.”	Literature review	Gen Z and their specific learning needs—learn by observation and practice (not by reading and listening), digital literacy lacking, visual learners, limited attention span and require adjustment of pedagogy. Discussion of the Digital Native—focuses on Generation Z (born late 1990s to early 2010s).
7.	Vitvitskaya et al., 2022 [53]	Behaviours and Characteristics of Digital Natives Throughout the Teaching-Learning Process: A Systematic Review	Peru	“The objective of this study was to systematize the scientific evidence on university teaching strategies related to the behaviour of digital natives and the characteristics of their learning.”	Systematic review	Systematic review of the behaviours and characteristics of digital natives in tertiary education. Provides extensive discussion of Digital Natives and their learning preferences. “Being part of the generation of digital natives does not necessarily mean having the skills to create content and publish in virtual environments. The myth is questioned, that there is a great disparity in literacy levels since although they remain hours connected and, on the network, there are many tools that are unknown in their daily practice.” Provides recommendations for the development of new strategies for teaching Digital Natives.
8.	Robb et al., 2014 [63]	Influential Factors and Perceptions of eHealth Literacy among Undergraduate College Students.	United States	1. What is the perceived eHealth literacy of undergraduate college students who have completed a required introductory college health and wellness course? 2. What personal and demographic factors influence perceived eHealth literacy in undergraduate college students? 3. What is the relationship between technology use and perceived eHealth literacy in undergraduate college students?	Survey	Students perceived that they knew how to use the Internet to answer questions about health but scored the lowest on confidence in using this information to make health decisions. These findings suggest that nursing faculty should consider ways to develop student eHealth literacy skills that will assist students in becoming confident informed consumers of eHealth information.
9.	Stec et al., 2018 [38]	Adaptation to a Curriculum Delivered via iPad: The Challenge of Being Early Adopters	United States	“The purpose of this research study is to determine how skills and attitudes in undergraduate and graduate students in a large, Midwestern university nursing program change when transitioning to the integration of iPads in the curriculum.”	Convergent mixed methods study	Study exploring the skills and attitudes towards iPad usage and whether this enhanced active learning. Discussion of the Digital Native: provides definition and brief discussion about the differences between student cohorts; including—“Recent work has questioned the relevance of the label ‘digital native’ and indicated that students’ experience and comfort level with a range of technologies is quite varied”.
10.	Zupanic et al., 2019 [48]	Media Use Among Students from Different Health Curricula: Survey Study	Germany	“The objective of this study was to explore whether there were differences in media use in students from various curricula at the Faculty of Health, Witten/Herdecke University.”	Cross-sectional study	Survey of media use in undergraduate health students (including nurses). Finds that individual curricula have different requirements for digital technology usage but do not take into account the different life circumstances of students. Examined similarities and differences between the cohorts of students. Discussion of Digital Native: focused on the fallacy of the Digital Native.
11.	Voge et al., 2012 [54]	The (Digital) Natives Are Restless: Designing and Implementing an Interactive Digital Media Assignment	United States	Description of a digital media assignment for undergraduate nursing students.	Pilot assignment design and evaluation	Discussion of piloting a digital media assignment with evaluation. “The National League for Nursing holds that educators are obligated to challenge their long-held traditions and design evidence based curricula that are flexible, responsive to students’ needs, collaborative, and integrate current technology”. Digitally interaction scenarios provide “another way to teach and learn in a dynamic discipline”. Discussion on the Digital Native: focuses on the need to respond to students’ learning needs.
12.	Vizcaya-Moreno et al., 2020 [39]	Social Media Used and Teaching Methods Preferred by Generation Z Students in the Nursing Clinical Learning Environment: A Cross-Sectional Research Study	Spain	“This cross-sectional research study aimed to explore the social media use and characteristics of Generation Z in nursing students and to identify what were the most useful and preferred teaching methods during clinical training.”	Cross-sectional study	Cross-sectional survey of Gen Z undergraduate nursing students regarding social media usage and preferred teaching methods on placement. Students identified social media use for personal rather than educational purposes. Students preferred “linking mentorship learning to clinical experiences, use of online tutorials or videos, interactive gaming, and virtual learning environments”. Extensive discussion of the differences between generational and their learning needs. Discussion of the Digital Native: “Generation Z nursing students have a distinctive combination of attitudes, beliefs, social norms, and behaviors that will modify education and the nursing profession”.
13.	Van Houwelingen et al., 2017 [43]	Internet-Generation Nursing Students’ View of Technology-Based Health Care	the Netherlands	“The aim of this study was to gain insight into today’s Internet-generation nursing students’ view of technology based health care and to determine whether the Internet generation believes that technology-based health care should be a part of nursing.”	Cross-sectional study	A survey of first-year nursing students to investigate students’ views on new health care technologies, with 28 activities presented with a short definition and students using a Likert scale to evaluate statements. Discussion of the Digital Native: includes the origins of the term with focus on Gen Z and states “According to generation rhetoric, one can argue that digital natives are already adequately equipped for this alternative type of care provision. However, this study shows the opposite and emphasizes the need for adequate telehealth technology education for all nurses, independent of their knowledge or lack of knowledge about the Internet”.
14.	Spencer 2012 [57]	Integrating Informatics in Undergraduate Nursing Curricula: Using the QSEN Framework as a Guide	United States	“This article uses the QSEN framework to present strategies for teaching multiple facets of informatics in the classroom, simulation laboratory, and clinical settings in a baccalaureate nursing curriculum.”	Literature review	Literature review on how to integrate nursing informatics into undergraduate nursing curricula, with a focus on the QSEN. Identifies that a barrier to the integration of NI into undergraduate nursing curricula is due to educators being “digital immigrants”. Identifies the important of knowledge, skills and attitudes in the adopting of nursing informatics.
15.	Skiba 2010 [52]	Digital Wisdom: A Necessary Faculty Competency?	United States	General discussion of the importance of digital literacy for educators.	Journal editorial	Text and opinion on the importance of digital literacy for faculty which focuses on the digital native and the origins of this term. Includes discussion of the debate regarding the Digital Native, and recommends moving to Prensky’s notion of “digital wisdom”. Identifies the issue of faculty not using digital technologies effectively in teaching and learning—“the next time you are considering the use of a technological tool, either in the physical or virtual classroom, think about how this tool will enhance the student’s ability to learn.”
16.	Skiba et al., 2006 [59]	Adapting Your Teaching to Accommodate the Net Generation of Learners	United States	“This article assists educators in teaching the Net Generation by highlighting the characteristics of the Net Generation and providing examples of how to adapt teaching strategies to accommodate the Net Generation, in light of their preferences for digital literacy, experiential learning, interactivity, and immediacy.”	Peer-reviewed publication	Text and opinion regarding adapting teaching to accommodate the Net Generation. “…the Net Generation requires a learner-centered model of education with a shift from the traditional teaching paradigm to a constructivist learning paradigm”. Significant discussion of digital literacy. Discussion of the Digital Native; includes Prensky’s definition with characteristics of the Net Generation, as described by Tapscott.
17.	Shorey et al., 2021 [51]	Learning styles, preferences and needs of generation Z healthcare students: Scoping review	Singapore	To consolidate evidence of the learning styles, preferences and needs of Generation (Gen) Z healthcare students.	Literature review	Gen Z healthcare students are characterized as digital natives who rely heavily on technology but have underdeveloped social skills and engage mainly in independent visual, sensing and active learning; Gen Z expect to be entertained with high-quality educational strategies that educators need to master and prepare.
18.	Sharoff 2011 [42]	Integrating YouTube into the Nursing Curriculum	United States	Discussion of using YouTube clips in nursing curriculum.	Text and opinion	We are only beginning to recognize the benefits of using YouTube in the classroom setting. The educational and instructive potential of YouTube is in its infancy. This highly interactive participatory teaching strategy is limitless in its potential for exciting students about learning. Nurse educators need to embrace engagement with social media tools as we work with students, patients, and the broader healthcare arena, working together to shape a healthier global community.
19.	Shamsaee et al., 2021 [58]	Assessing the effect of virtual education on information literacy competency for evidence-based practice among the undergraduate nursing students	Iran	Interventional study	Educational intervention	Pre and post survey showed that virtual education had a significant effect on information-seeking skills and knowledge about search operators in nursing students. Nurse educators can benefit from our experiences in designing this method for the use of virtual education programs in nursing schools.
20.	Pieterse et al., 2018 [64]	A Multicultural Approach to Digital Information Literacy Skills Evaluation in an Israeli College	Israel	This study reports findings from first-year students’ self-estimation of their information skills according to two information literacy models.	Survey	The researchers found that native Hebrew- speaking students preferred digital sources, while students with Hebrew as second language (Arabic-speaking) preferred printed sources, and both groups ranked their technological and information literacy skills as above average. The study supports previous research on the Arabic-speaking students’ need for more mediation in the dimensions of information literacy examined compared to Hebrew-speaking students, despite no significant difference in access to the internet at home and self-assessment of their general computing skills.
21.	Orkiszewski et al., 2016 [41]	Reaching Millennials with Nursing History	United States	Discussion of the North Carolina Nursing History website and its role in educating nursing students regarding the historical context of nursing.	Scholarship of teaching	Discussion of a history website that links with the learning needs of Millennials for digital information. Links understanding of nursing history with historical literacy. “a significant need for educators to better understand generational learners and to recognize an imbalance between students’ expectations of the learning environment and the actual environment” were identified. Discussion of the Digital Native: focuses on Prensky’s definition and links with the characteristics of Millennials.
22.	Nsouli et al., 2021 [37]	Attitudes of nursing faculty members toward technology and e-learning in Lebanon	Lebanon	“A mixed methodological research approach was used to investigate the attitudes of nursing teaching staff toward the use of ICT in nursing education.”	Mixed methods	Mixed-method study exploring attitudes of faculty towards technology and eLearning. Addresses the sociopolitical structure of Lebanon and its impact on ICT adoption. It is “…a necessity to support clinicians in gaining experience in digital health approaches, and nurture the career pathways of those who show an early interest”. Identifies gaps in undergraduate nursing curricula and the lack of informatics education. “This study further elaborated that stress, lack of experience, lack of knowledge, limited skills, and poor infrastructure, are factors that prevent educators from using ICT in their teaching practices.” Identifies three faculty groups—pioneers, followers and resisters. Discussion of the Digital Native: links with Prensky and the Digital Native.
23.	Mather et al., 2022 [47]	eHealth Literacy of Australian Undergraduate Health Profession Students: A Descriptive Study	Australia	“The aim of this study was to explore the eHealth literacy of undergraduate health profession students to inform undergraduate curriculum development to promote work-readiness.”	Exploratory descriptive study	Exploratory study of the eHealth literacy of undergraduate health professional students, with an extensive definition of eHealth literacy. Suggestion that digital health skills should be integrated into undergraduate curriculums. Provides extensive implications for education curricula. Not a definition of the Digital Native, but a valid source as it discusses the differences in confidence in the use of digital technologies associated with age.
24.	Johnson 2018 [49]	Success in information technology—what do student nurses think it takes? A quantitative study based on Legitimation Code Theory	UK	In one UK university to find out what approach to learning they thought would lead to success in IT.	Quantitative survey	Not everyone is “good at IT”; third year students agreed they need to know more about IT and that certain “types of people are better at IT than others” Nurse academics to nurture aptitudes, attitudes and dispositions, perhaps through course design that embeds discipline-specific use of IT, promoting digital fluency as a side effect of focusing on epistemic fluency in the design of learning activities.
25.	Hills et al., 2017 [46]	Generation Y Health Professional Students’ Preferred Teaching and Learning Approaches: A Systematic Review	Ireland Australia	“The aim of this systematic review is to present the best available evidence on teaching and learning strategies or methods preferred by ‘Generation Y’ health care professional students.”	Systematic Review	Systematic review of the preferred teaching and learning needs of Gen Y students—only five studies included in extraction. Discusses teaching approaches—lecture, group work, lecture versus group work, self-directed learning, web-based learning, case stories and case studies, teaching clinical skills, technology and visual aids, classroom structure and community service. “While generational profiles have been used as a framework for investigating the teaching and learning preferences common to each generation, the results of this review neither confirm nor refute taking a generational perspective to explore teaching and learning preferences.” Discussion of the Digital Native: defines Gen Y (and other generations), provides counter-argument against Prensky’s definition of the digital native, discusses and critiques generational preferences for use of technologies and teaching methods.
26.	Earle et al., 2009 [60]	Nursing Pedagogy and the Intergenerational Discourse	Canada	“This article examines the effects of intergenerational diversity on pedagogical practice in nursing education and highlights the need for nurse educators to engage in a critical discourse regarding the adequacy of current pedagogy in fostering an ethos that can optimize the teaching-learning process and promote ongoing learning for the future.”	Literature review	Discussion of viewing education through a “generational lens” and whether nursing curricula is meeting undergraduate nursing students’ needs. Assertion that “the majority of students in today’s university and college classrooms belong to the technologically savvy Millennial Generation. These learners are described as assertive, optimistic, self-reliant, and inquisitive.” Identifies unique learning styles of millennials and need to accommodate them to attract more students to nursing. Discussion of the Digital Native: focuses on generational similarities and differences between Gen X, Gen Y and Gen Z.
27.	Christodoulou et al., 2015 [32]	The Test—Retest Reliability and Pilot Testing of the “New Technology and Nursing Students’ Learning Styles” Questionnaire	Greece	“To estimate the validity and reliability of an assessment tool designed to identify the undergraduate nursing students’ digital literacy as well as their learning preferences”.	Non-randomised experimental study	Discussion of the Net Generation or Millennials—“millennials have different preferences and style of learning and thus many challenges have been posed to the educational institutes”. Testing of a questionnaire to evaluate technology and learning styles of new students. Discussion of the Digital Native: detailed discussion of the Digital Native and Digital Native debate.
28.	Bembridge et al., 2010 [55]	The preparation of technologically literate graduates for professional practice	Australia	“…o examine the transferability of the ICT skills acquired at university to contemporary practice environments”	Literature review	Discussion paper, including a historical overview, of preparing digitally literate nursing graduates in Australia. Gaps between ICT in education and ICT in clinical practice. Need for digital competency to function in the workplace. Lack of understanding by students of the role of digital technologies in patient care. Lack of ICT competency standards for undergraduate education. Need for “basic and specialised ICT skills that cannot be met by generic ICT training courses”. Discussion of the Digital Native: linked with technologically literate graduates.
29.	Atkey et al., 2020 [36]	What do Nursing Students’ Stories Reveal about the Development of their Technological Skills and Digital Identity? A Narrative Inquiry	Canada	“This narrative qualitative study aims to explore nursing students’ development of their technological skills and digital identities by assembling an unbiased collection of narrative stories. Specifically, this study will use a narrative framework to ask what nursing students’ stories reveal about their technological skills and the development of professional digital identities.”	Qualitative research	An understanding of digital technologies is essential for nursing students. Links Prensky with how students learn but cautions this is only one of the influencing factors. Identifies both the benefits and risks of technology. Provides narratives from students on their experiences with digital technologies. Provides recommendations for further research. Discussion of the Digital Native: linked with professional digital identity, loss of teaching traditions and risk of students lacking ability to engage with patients effectively.
